# Genetic investigation of the contribution of body composition to anorexia nervosa in an electronic health record setting

**DOI:** 10.1038/s41398-022-02251-y

**Published:** 2022-11-19

**Authors:** Taralynn Mack, Sandra Sanchez-Roige, Lea K. Davis

**Affiliations:** 1 Department of Medicine, Division of Genetic Medicine, Vanderbilt University Medical Center, Nashville, TN 37232 USA; 2 Vanderbilt Genetics Institute, Vanderbilt University Medical Center, Nashville, TN USA; 3 Department of Psychiatry, University of California San Diego, La Jolla, CA 92093 USA; 4 Department of Biomedical Informatics, Vanderbilt University Medical Center, Nashville, TN USA; 5 Department of Psychiatry and Behavioral Sciences, Vanderbilt University Medical Center, Nashville, TN USA; 6 Department of Molecular Physiology and Biophysics, Vanderbilt University, Nashville, TN USA

**Keywords:** Psychiatric disorders, Scientific community

## Abstract

Anorexia nervosa (AN) is a psychiatric disorder defined by anthropometric symptoms, such as low body weight, and cognitive-behavioral symptoms, such as restricted eating, fear of weight gain, and distorted body image. Recent studies have identified a genetic association between AN and metabolic/anthropometric factors, including body mass index (BMI). Although the reported associations may be under pleiotropic genetic influences, they may represent independent risk factors for AN. Here we examined the independent contributions of genetic predisposition to low body weight and polygenic risk (PRS) for AN in a clinical population (Vanderbilt University Medical Center biobank, BioVU). We fitted logistic and linear regression models in a retrospective case-control design (123 AN patients, 615 age-matched controls). We replicated the genetic correlations between PRS_BMI_ and AN (*p* = 1.12 × 10^−3^, OR = 0.96), but this correlation disappeared when controlling for lowest BMI (*p* = 0.84, OR = 1.00). Additionally, we performed a phenome-wide association analysis of the PRS_AN_ and found that the associations with metabolic phenotypes were attenuated when controlling for PRS_BMI_. These findings suggest that the genetic association between BMI and AN may be a consequence of the weight-related diagnostic criteria for AN and that genetically regulated anthropometric traits (like BMI) may be independent of AN psychopathology. If so, individuals with cognitive-behavioral symptomatology suggestive of AN, but with a higher PRS_BMI_, may be under-diagnosed given current diagnostic criteria. Furthermore, PRS_BMI_ may serve as an independent risk factor for weight loss and weight gain during recovery.

## Introduction

Anorexia nervosa (AN) is amongst the psychiatric disorders with the highest mortality rates [1]. According to the Diagnostic and Statistical Manual of Mental Disorders (DSM), the symptomatology of AN is diverse, including anthropometric symptoms, such as low body weight, and cognitive-behavioral symptoms, such as a strong fear of weight gain or continuous behavior to avoid weight gain, and body image/self-worth contingent on physical appearance [2]. The etiology of AN is multifactorial, with substantial environmental and genetic influences [1, 3–7]. AN is heritable, with SNP and twin heritability estimates ranging from 20% to 58%, respectively [1, 8, 9]. Understanding the genetic basis for each of the AN symptom domains could improve diagnosis and treatment mechanisms.

Under the earlier diagnostic criteria of the DSM-IV, an AN diagnosis could only be given if the patient was clinically underweight [10]. Since the implementation of the DSM-5 [2], those criteria were modified to “a significantly low body weight in the context of age, sex, developmental trajectory, and physical health”. Nonetheless, low body weight remains a hallmark of the disorder. The criterion for low body weight can make it difficult for patients to receive a diagnosis, and therefore treatment if they exhibit other symptoms of the disorder but do not have significantly low body weight. In some cases, individuals with subsyndromal forms of AN presenting with a normal or above normal body mass index (BMI) may be diagnosed with atypical AN. Compared to individuals with AN diagnosis, these atypical AN patients usually have a longer duration of symptoms and greater weight loss before diagnosis [11], and are less likely to receive inpatient treatment for their condition [12].

In addition to the role of low body weight as diagnostic criteria, emerging genetic studies have identified a possible metabolic component to genetic risk for anorexia [1, 13, 14]. For instance, AN is negatively genetically correlated with BMI [15], triglyceride levels, and fasting insulin, and positively correlated with metabolic markers like HDL cholesterol [16]. Furthermore, BMI heritability is heavily concentrated in the tissues of the central nervous system, which are also directly involved in the cognitive and behavioral aspects of anorexia [17], leading to the hypothesis that metabolic factors are involved in the development of AN [18]. However, questions regarding the nature of the relationship between metabolic control and AN, remain. It is hypothesized that AN and anthropometric factors, such as BMI, may share a common genetic basis due to the pleiotropic effects of genes that simultaneously influence both phenotypes. Alternatively, it is also possible that the observed association between PRS_BMI_ and AN diagnosis is in part due to the weight-related diagnostic criteria for anorexia. For example, because the GWAS for AN is reliant on diagnostic criteria requiring a low BMI, it is possible that BMI is acting as a collider variable [19], potentially inducing genetic correlations between AN and BMI. If true, then genetic correlates of BMI may not be involved in AN psychopathology per se, but may instead be critical for understanding the extent and rate of weight loss that can occur as a consequence of AN. For example, patients in treatment for AN often return to very low weights even following full recovery, which may also be, in part, explained by genetic variations that contribute to increased metabolism and a lower genetic “set-point” for body weight [20]. Therefore, in the present study, we set out to illuminate the genetic association between AN and BMI using health record data over time from cases and controls collected in a biobank setting.

## Materials and methods

### Study population and anorexia phenotype

Our cohort included 123 genotyped female subjects from the Vanderbilt University Medical Center biobank (BioVU) with a lifetime diagnosis of AN as determined via at least one ICD-9 (307.1) or ICD-10 (F50*) codes for AN. BioVU is a repository of leftover blood samples (~240,000 samples) from clinical testing, which are sequenced, de-identified, and linked to clinical and demographic data [21]. All BioVU participants have provided informed consent. The VUMC Institutional Review Board oversees BioVU and approved this project (IRB#160302).

The prevalence of AN in the BioVU population was ~0.65%, which is similar to that observed in the general population (~0.9%) [22]. Control subjects (*N* = 615) were identified as those without ICD codes for AN. A ratio of 5:1 controls to cases was used, as the effect of increased statistical power is negligible above that ratio [23]. We restricted the sample to females due to the very small number of male AN cases with genotype data available (*N* = 2).

We assessed the relationship between AN genetic risk, and both mean and lowest BMI, as mean BMI better summarizes long-term BMI over lifetime, while lowest BMI better reflects biological extremes. We defined two separate control samples to test hypotheses related to mean and lowest BMI, each containing 615 female subjects (1:5 ratio) without the ICD-9 or ICD-10 codes for AN. The first set was age-matched to cases based on the median age across the medical record for each individual for use in analyses involving mean BMI. Median age is used to represent the age of the individual while they were a patient at VUMC. The second control set was age-matched to cases based on the age at lowest recorded BMI for use in analyses involving lowest BMI. Because lowest BMI is a single incident measurement, the corresponding age at lowest BMI is the most appropriate age variable.

### Calculation of BMI

For every individual in BioVU, age and BMI measurements were collected from their de-identified EHR. After quality controls (QC, see Supplementary Materials), mean BMI and lowest BMI were calculated for each individual.

### Generation of polygenic scores

We calculated polygenic risk scores (PRS) using PRC-CS [24] “auto” version (i.e., the global shrinkage parameter phi was learned from the data in a Bayesian approach) for each of the defined AN groups, as well as for each of the 66,914 BioVU individuals genotyped on the Illumina MEGA-EX array for further exploratory analyses. Genotyping and QC of this sample have been described elsewhere [21, 25]. We used GWAS summary statistics for AN from the largest available study (*N* = 72,517) [1]. For BMI, we used the female stratified GWAS summary statistics from the GIANT Consortium and UK BioBank meta-analysis (*N* ~430,000) [26]. Scores were *z* score standardized for both PRS_AN_ and PRS_BMI_.

### Statistical analyses

We tested a total of eight multivariable regression models, including four logistic regression models and four linear regression models. The first ten principal components calculated from the genetic data were included in all models to control for residual population stratification. To account for multiple testing, we used a Bonferroni corrected *p* value of 6.25 × 10^−3^ (0.05/8) to determine statistical significance.

We first examined if the PRS_AN_ was significantly associated with the diagnosis of AN; and if PRS_BMI_ was significantly associated with both mean and lowest BMI. We then hypothesized that PRS_AN_ would be associated with mean lifetime BMI and with lowest BMI, regardless of whether the diagnosis of anorexia is present. To test this hypothesis, we regressed PRS_AN_ on mean BMI and, separately, on lowest BMI, while controlling for AN diagnosis.

Next, we tested whether the PRS_BMI_ variable was associated with AN diagnosis, and then assessed whether that association remained after controlling for the lowest measured BMI. Similarly, we also investigated the effect of including a covariate for the lowest BMI when regressing PRS_AN_ on the AN diagnosis.

### Mediation analysis

We performed two mediation analyses, first we tested a model in which PRS_BMI_ was the exposure, lowest BMI was the mediator, and AN diagnosis was the outcome. Second, we tested a model in which PRS_AN_ was the exposure, lowest BMI was the mediator, and AN diagnosis was the outcome. Bootstrapping (10,000 iterations) was used to generate confidence intervals and determine statistical significance. The analyses were performed using the *mediation* R package v4.5.0 [25]. Due to the limitations of EHR data, we were unable to conclusively determine the chronological order of lowest BMI measurement and AN diagnosis.

### PheWAS analyses

In the BioVU sample (*N* = 66,914), we fitted a logistic regression for each of the 1335 disease phenotypes available to estimate the odds of a diagnosis of that phenotype given the PRS_AN_. Each disease phenotype (commonly referred to as “phecode”; https://phewascatalog.org/phecodes, Phecode Map 1.2) was classified using EHR and ICD diagnostic codes to establish “case” status. For an individual to be considered a case, they were required to have two separate ICD codes for the index phenotype, and each phenotype needed at least 100 cases to be included in the analysis.

We performed an exploratory phenome-wide association analysis (PheWAS) to examine genetic associations between PRS_AN_ and thousands of other phenotypes in the medical phenome, including metabolic conditions. We repeated the analyses after adjusting for PRS_BMI_ to determine the impact of genetic correlates of BMI on these associations. The covariates included in the analyses were sex, median age of the longitudinal EHR measurements, and the top ten principal components of ancestry. We repeated the analyses including AN diagnosis and PRS_BMI_, respectively, as additional covariates. We used the standard Benjamini–Hochberg false discovery rate (FDR 5%) to correct for multiple testing. PheWAS analyses were run using the PheWAS R package v0.12 [27].

## Results

### BMI distributions and sample characteristics

Mean and lowest BMI was significantly lower among cases compared to controls (cases (mean, SD) = 20.93, 4.47; (lowest, SD) = 18.14, 4.31; controls (mean, SD) = 26.27, 6.81; (lowest, SD) 20.70, 6.48). Because the groups were age-matched, the average median age for both cases and controls was 26 (range 11–79), and the average age at the lowest BMI was also 26 (range 12–78).

In the case group, 65% of individuals had an underweight lowest BMI at some point in their BioVU medical record, while only 13% of controls had an underweight lowest BMI. Additionally, 82% of cases had a mean BMI that was normal or below, while only 48% of controls had a mean BMI that was normal or below normal.

### Regression models

**Fig. 1:**
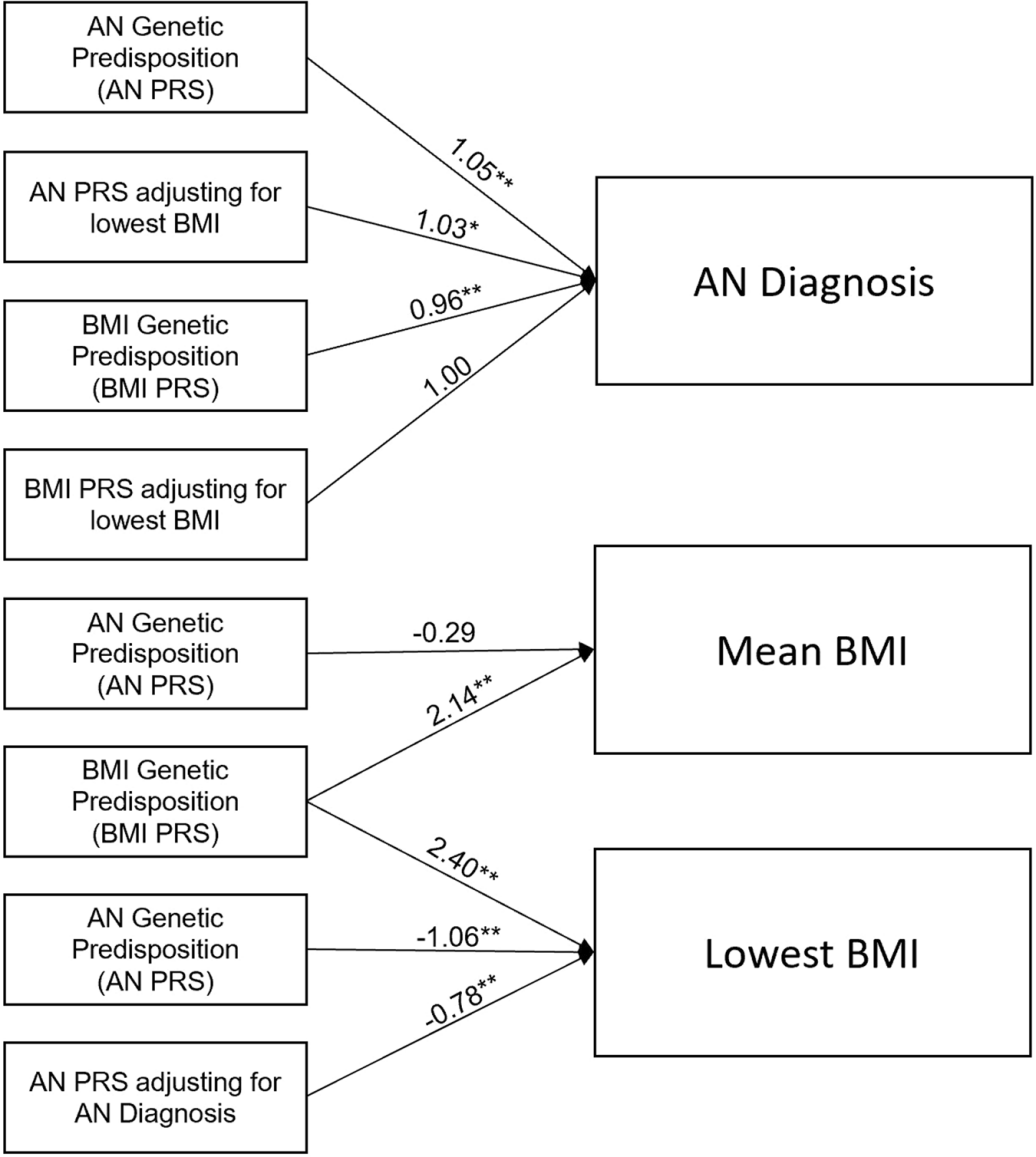
Schematic representation of the associations tested. Arrows between boxes denote associations tested. Beta values and significance (**p < 6.25 × 10^−3^) (*p < 0.05) are reported for associations with mean/lowest BMI, while odds ratios and significance are reported for associations with AN diagnosis.

Figure 1 presents the results for all the tested regression models. As expected, PRS_AN_ was associated with AN diagnosis (*p* = 6.25 × 10^−4^, OR = 1.05, CI = 0.97, 1.07). PRS_BMI_ was associated with mean BMI (*p* = 2.00 × 10^−16^, *β* = 2.14, SE = 0.23) and with lowest BMI (*p* = 2.00 × 10^−16^, *β* = 2.40, SE = 0.23).

PRS_AN_ was not associated with mean BMI (*p* = 0.88, *β* = −0.001, SE = 0.01) but was associated with lowest BMI (*p* = 9.03 × 10^−6^, *β* = −0.03, SE = 0.01), even after controlling for AN diagnosis (*p* = 6.46 × 10^−4^, *β* = −0.02, SE = 0.01). As previously reported by others, we observed a negative association between PRS_BMI_ and AN diagnosis (*p* = 1.12 × 10^−3^, OR = 0.96, SE = 0.01). After accounting for the lowest measured BMI, the association between PRS_AN_ and AN diagnosis remained nominally significant (*p* = 0.01, OR = 1.03, SE = 0.01) indicating the PRS_AN_ contributes to the AN diagnosis beyond contributions to body weight. In contrast, after controlling for lowest measured BMI, the association between PRS_BMI_ and AN diagnosis was null (*p* = 0.84, OR = 1.02, SE = 0.01).

Using a mediation model, we found that nearly the entire effect of the PRS_BMI_ on AN diagnosis was accounted for by the lowest BMI measured (proportion variance mediated = 95%, *p* = 2.00 × 10^−16^). Again, in contrast, the lowest measured BMI only accounted for 40% of the variance (*p* = 3.24 × 10^−3^) in AN diagnosis contributed by the PRS_AN_.

### PheWAS results

**Fig. 2:**
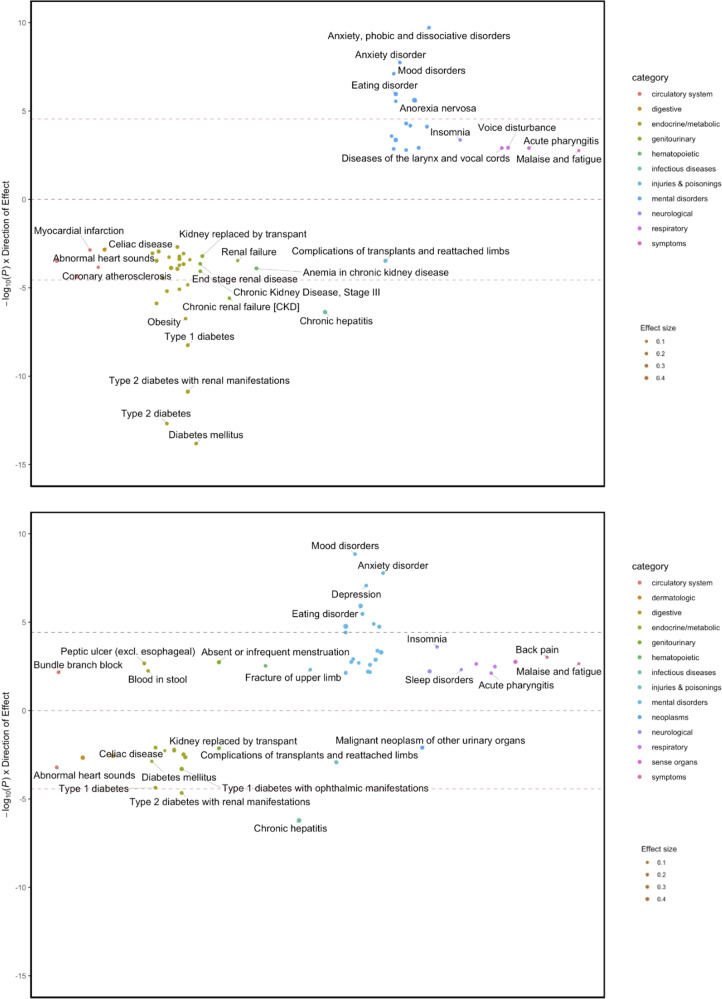
PheWAS analysis showing shared genetic associations between risk for anorexia nervosa and other phenotypes in females. Significant positive correlations are shown between psychiatric conditions and significantly negative correlations are shown with metabolic phenotypes (upper panel). After controlling for PRS_BMI_, most of the metabolic phenotypes were attenuated (lower panel), while the psychiatric phenotypes remained relatively unchanged.

PRS_AN_ was initially significantly associated with metabolic and psychiatric phenotypes, including positive associations with anxiety (*β* = 0.07, *p* = 1.81 × 10^−8^) and mood disorders (*β* = 0.07, *p* = 7.81 × 10^−8^) and negative associations with obesity and diabetes (*β* = −0.08, *p* = 1.80 × 10^−7^, *β* = −0.09, *p* = 1.53 × 10^−14^, respectively; Fig. 2, Supplementary Table 1), even when controlling for AN diagnosis (Supplementary Table 2). However, when PRS_BMI_ was included as a covariate, the magnitude and strength of the associations with metabolic phenotypes were substantially decreased (Fig. 2, Supplementary Table 3), suggesting that these initial observations were largely a consequence of the genetic association between the PRS_AN_ and the PRS_BMI_.

## Discussion

Here, we investigated the role of measured BMI as a mediator in the observed genetic relationship between AN and metabolic factors. Recent genetic studies suggest that AN should be recategorized as a metabo-psychiatric disorder due to the observed genetic associations between risk for AN and metabolic factors [1, 13, 18]. The present study does not suggest the complete absence of a link between AN and all metabolic factors, but proposes that there should be less emphasis on body weight as a diagnostic criterion as measured BMI mediates a significant portion of this link.

We provide evidence that suggests that the association between AN and anthropometric factors is potentially driven by the genetic predisposition of an individual to present with a low body weight (but that the PRS_BMI_ is not necessarily involved in other aspects of AN symptomatology). In other words, PRS_BMI_ may contribute to the AN diagnosis by decreasing the body’s “set-point” BMI, thus increasing the likelihood that extremely low body weight will be observed in addition to the other AN symptoms, thereby increasing odds of receiving an AN diagnosis. This finding has two important implications. First, it suggests that individuals with cognitive-behavioral symptomatology suggestive of AN, but with a higher genetically predicted BMI, may be under-diagnosed given current diagnostic criteria. Second, it suggests that the PRS_BMI_ may be an important *independent* risk factor in the life-threatening consequences of extremely low BMI in the context of AN.

We found that PRS_AN_ and PRS_BMI_ were strongly associated with AN diagnosis and measured BMI, respectively, highlighting the validity of the EHR for genetic analysis for both traits. Intriguingly, PRS_AN_ was not associated with *mean* BMI but was associated with lowest BMI, suggesting that some of the genetic factors involved in the diagnosis of AN may also contribute (behaviorally or otherwise) to loss of body weight [1], even in individuals who do not have a diagnosis of AN.

Both PRS_BMI_ and PRS_AN_ were associated with AN diagnosis, as previously speculated, but in this study, we were able to further dissect the contributions of PRS_BMI_. While initial results showed that PRS_BMI_ was associated with AN diagnosis, the conditional analysis demonstrated that PRS_BMI_ was not associated with AN diagnosis outside of its effect on BMI. In contrast, PRS_AN_ remained robustly associated with AN diagnosis even after adjusting for the lowest BMI. We speculate that these results suggest that the PRS_BMI_ mostly contributes to the severe consequences of AN (low body weight), which often bring people to clinical attention, while the PRS_AN_ may represent an increased risk for the cognitive behavioral processes that lead to the development of AN. Analyses from our mediation analyses showed that the lowest BMI almost entirely accounted for the association between PRS_BMI_ and AN diagnosis, but only partially accounted for the association between PRS_AN_ and AN diagnosis. This is further evidence for our hypothesis that individuals with higher PRS_BMI_ may be underdiagnosed given the current diagnostic criteria for AN. These findings are consistent with a recent study that did not find any significant associations between eating disorder symptoms and metabolic PRSs, suggesting that the metabolic genetic factors could distinguish between symptoms of disordered eating and a clinical eating disorder diagnosis [28]. There is a well-documented association between PRS_BMI_ and disordered eating behaviors, which has been shown to be mediated by measured BMI. Individuals with higher PRS_BMI_ show both higher measured BMI and weight loss behaviors [29]. Additionally, higher PRS_AN_ has been linked to weight loss trajectory in individuals without a clinical diagnosis of AN, and this association is not mediated through genetic risk for obesity, which likely has shared genetic architecture with PRS_BMI_ [30].

In the PheWAS analyses, PRS_AN_ was associated with numerous health outcomes, and metabolic conditions were strongly implicated. This replicates and augments recent evidence showing positive correlations for AN with psychiatric phenotypes and negative correlations with diabetes and metabolism phenotypes [1, 13, 16, 18]. Chronic hepatitis and chronic renal failure were also significantly associated with the PRS_AN_, as these conditions are associated with poor appetite [31, 32]. However, when we controlled for genetically predicted BMI (i.e., PRS_BMI_), the associations between PRS_AN_ and metabolic factors significantly weakened. This is in contrast to a previous study that did not observe significant attenuation of the correlation between genetic associations of metabolic factors and AN when controlling for genetic associations of BMI [1]. Our findings further signal that the association between genetic risk for AN and metabolic outcomes is potentially largely attributable to BMI, which is difficult to disentangle since low BMI is a diagnostic criterion of AN. This is important because metabolic dysregulation in individuals with AN and low BMI may further increase difficulty in maintaining a healthy BMI [1].

In addition to phenotypes directly related to AN symptomatology, other phenotypes continued to be associated with PRS_AN_ after adjustment for PRS_BMI_ in the PheWAS analysis. Notably, chronic hepatitis was negatively associated with PRS_AN_. While reversible severe hepatitis is sometimes observed in severe AN [33], chronic hepatitis is novel. Additionally, fractures and back pain were positively associated with PRS_AN_, which may reflect both increased physical activity and decreased bone density observed in AN patients [34]. Further studies are needed to investigate these associations and their role in the genetic architecture of AN.

These findings of decreased metabolic condition correlations when controlling for BMI are consistent with a recent study that stratified individuals into high, normal, and low BMI groups and performed three separate PheWAS analyses [35]. There were observed differences in the association between AN and BMI, with the strongest negative association in the high BMI group, demonstrating that measured BMI plays a role. However, our study controlled for PRS_BMI_ rather than measured BMI, which allowed us to dissect the components of AN risk that are solely due to *genetic* body composition differences between individuals. This means that the collider effect of BMI on the correlation between AN risk and metabolic conditions extends farther than weight bias and extends into the realm of entangled genetic etiology.

Although the AN underweight diagnostic criteria were recently expanded upon the release of the DSM-5 in 2013, low body weight still remains a hallmark feature. Our findings emphasize that there needs to be a shift away from body weight as an important diagnostic criterion for AN, particularly in individuals with subsyndromal forms of AN (atypical AN), because it can lead to underdiagnosis and makes it difficult to disentangle the true genetic contributions to AN. Instead, low PRS_BMI_ should be used to predict an increased risk of being underweight while exhibiting AN symptoms, consistent with research showing individuals with high PRS_AN_ and low PRS_BMI_ had significantly slower growth trajectories than those with high PRS_AN_ and PRS_BMI_ [36]. Additionally, results from a recent study suggest that the use of PRS_BMI_ in addition to PRS_AN_ could be a useful method to predict individuals that will develop severe and enduring eating disorders [37].

These ideas have been shown in clinical settings. A recent study on hospitalizations from AN and atypical AN (non-underweight patients) found that patients displayed similar medical complications regardless of weight and that duration of illness was a much stronger predictor for severity than body weight [38]. When present alongside AN behaviors, weight suppression of even five percent can be clinically significant [39]. Atypical AN occurs in up to 3% of the population, meaning that these “atypical” individuals represent the majority of cases by far [40]. In fact, under the DSM-IV, over half of all patients diagnosed with an eating disorder were given an ED-NOS (Eating Disorder Not Otherwise Specified) diagnosis due to the absence of one or more of the stringent criteria for the established disorders [41]. This may bias AN diagnoses towards only those with the most extreme BMI manifestations of the disease [42]. Patients with the same severity of symptoms who present at higher weights may not be able to receive insurance coverage/treatment due to inherent bias among health professionals [43, 44]. We predict that risk scores for AN will improve in the future if the focus continues to shift away from low body weight, due to the fact that more cases will be identified.

It is worth acknowledging the limitations of our study. First, larger sample sizes are still needed as well as provide validation across other populations and sample section schemes. For example, it is possible that the observed trends are unique to AN diagnosis in an academic medical center and may be different in a community care setting [45]. Additionally, this study focused on individuals of European ancestry leaving a research gap that needs to be filled with studies from diverse populations [46, 47]. The use of ICD codes as a diagnostic tool is also challenging. Because ICD codes are primarily used for billing, they do not always serve as an accurate predictor of a patient’s specific medical diagnosis [48]; however, we have shown strong genetic correlations between ICD codes and clinical diagnosis, and note that ICD codes were also used to define cases in the recent large GWAS of AN [1]. Lastly, it is difficult to assess the true BMI history of an individual through medical records. Lowest BMI based on a lifetime lowest BMI may not necessarily represent the BMI during the active illness period. Therefore, more comprehensive, or ideally, prospective studies on BMI that involve more frequent measurements would be an improvement [49, 50]. Gathering longitudinal data and performing a mediation analysis taking the underlying timeline into account may help us further examine whether PRS_BMI_ significantly contributes to AN beyond its effects on BMI. Future GWAS of AN symptomatology would allow us to test whether PRS_BMI_ is associated with low body weight over the other symptoms.

Overall, there is a clear relationship between AN diagnosis and body composition. Our work speaks to the importance of exploring potential hypotheses to explain this complex relationship.

## Supplementary information

Supplementary Text

Supplementary Table

Supplementary Table 1

Supplementary Table 2

Supplementary Table 3

## References

[1] Watson HJ, Yilmaz Z, Thorton L, Hübel C, Coleman JRI, Gaspar HA (2019). Genome-wide association study identifies eight risk loci and implicates metabo-psychiatric origins for anorexia nervosa. Nat Genet.

[2] American Psychiatric Association. Diagnostic and statistical manual of mental disorders: DSM-5. American Psychiatric Association, 2013.

[3] Wang K, Zhang H, Bloss CS, Duvvuri V, Kaye W, Schork NJ (2011). A genome-wide association study on common SNPs and rare CNVs in anorexia nervosa. Mol Psychiatry.

[4] Zipfel S, Giel KE, Bulik CM, Hay P, Schmidt U (2015). Anorexia nervosa: aetiology, assessment, and treatment. Lancet Psychiatry.

[5] Culbert KM, Racine SE, Klump KL (2015). Research review: what we have learned about the causes of eating disorders - a synthesis of sociocultural, psychological, and biological research. J Child Psychol Psychiatry.

[6] Paolacci S, Kiani AK, Manara E, Beccari T, Ceccarini MR, Stuppia L (2020). Genetic contributions to the etiology of anorexia nervosa: New perspectives in molecular diagnosis and treatment. Mol Genet Genom Med..

[7] Treasure J, Zipfel S, Micali N, Wade T, Stice E, Claudino A (2015). Anorexia nervosa. Nat Rev Dis Prim.

[8] Thornton LM, Mazzeo SE, Bulik CM (2011). The heritability of eating disorders: methods and current findings. Curr Top Behav Neurosci I.

[9] Mayhew AJ, Pigeyre M, Couturier J, Meyre D (2018). An evolutionary genetic perspective of eating disorders. Neuroendocrinology.

[10] American Psychiatric Association. Diagnostic and statistical manual of mental disorders: DSM-IV. American Psychiatric Association, 1994.

[11] Lebow J, Sim LA, Kransdorf LN (2015). Prevalence of a history of overweight and obesity in adolescents with restrictive eating disorders. J Adolesc Health.

[12] Kennedy GA, Forman SF, Woods ER, Hergenroeder AC, Mammel KA, Fisher MM (2017). History of overweight/obesity as predictor of care received at 1-year follow-up in adolescents with anorexia nervosa or atypical anorexia nervosa. J Adolesc Health Publ Soc Adolesc Med..

[13] Duncan L, Yilmaz Z, Gaspar H, Walters R, Goldstein J, Anttila V (2017). Significant locus and metabolic genetic correlations revealed in genome-wide association study of anorexia nervosa. Am J Psychiatry.

[14] Hübel C, Gaspar HA, Coleman JRI, Finucane H, Purves KL, Hanscombe KB (2019). Genomics of body fat percentage may contribute to sex bias in anorexia nervosa. Am J Med Genet Part B Neuropsychiatr Genet..

[15] Anttila V, Bulik-Sullivan B, Finucane H, Walters RK, Bras J, The Brainstorm Consortium (2018). Analysis of shared heritability in common disorders of the brain. Science.

[16] Yilmaz Z, Halvorsen M, Bryois J, Yu D, Thorton LM, Zerwas S (2020). Examination of the shared genetic basis of anorexia nervosa and obsessive-compulsive disorder. Mol Psychiatry.

[17] Shungin D, Winkler TW, Croteau-Chonka DC, Ferreira T, Locke AE, Mägi R (2015). New genetic loci link adipose and insulin biology to body fat distribution. Nature.

[18] Bulik CM, Carroll IM, Mehler P (2021). Reframing anorexia nervosa as a metabo-psychiatric disorder. Trends Endocrinol Metab..

[19] Munafò MR, Tilling K, Taylor AE, Evans DM, Davey, Smith G (2018). Collider scope: when selection bias can substantially influence observed associations. Int J Epidemiol..

[20] Mustelin L, Raevuori A, Bulik CM, Rissanen A, Hoek HW, Kaprio J (2015). Long-term outcome in anorexia nervosa in the community. Int J Eat Disord.

[21] Roden DM, Pulley JM, Basford MA, Bernard GR, Clayton EW, Balser JR (2008). Development of a large-scale de-identified DNA biobank to enable personalized medicine. Clin Pharmacol Ther..

[22] Smink FRE, van Hoeken D, Hoek HW (2012). Epidemiology of eating disorders: incidence, prevalence and mortality rates. Curr Psychiatry Rep..

[23] Kuo CL, Duan Y, Grady J. Unconditional or conditional logistic regression model for age-matched case–control data? Front. Public Health 2018;6:57. 10.3389/fpubh.2018.00057 PMC5840200 29552553

[24] Ge T, Chen CY, Ni Y, Feng YCA, Smoller JW (2019). Polygenic prediction via Bayesian regression and continuous shrinkage priors. Nat Commun.

[25] Dennis J, Sealock J, Levinson RT, Farber-Eger E, Franco J, Fong S (2021). Genetic risk for major depressive disorder and loneliness in gender-specific associations with coronary artery disease. Mol Psychiatry.

[26] Yengo L, Sidorenko J, Kemper KE, Zheng Z, Wood AR, Weedon MN (2018). Meta-analysis of genome-wide association studies for height and body mass index in ∼700000 individuals of European ancestry. Hum Mol Genet..

[27] Carroll RJ, Bastarache L, Denny JCR (2014). PheWAS: data analysis and plotting tools for phenome-wide association studies in the R environment. Bioinformatics..

[28] Abdulkadir M, Hübel C, Herle M, Loos RJF, Breen G, Bulik CM (2022). Eating disorder symptoms and their associations with anthropometric and psychiatric polygenic scores. Eur Eat Disord Rev J Eat Disord Assoc..

[29] Abdulkadir M, Herle M, De Stavola BL, Hübel C, Santos Ferreira DL, Loos RJF (2020). Polygenic score for body mass index is associated with disordered eating in a general Population Cohort. J Clin Med..

[30] Xu J, Johnson JS, Signer R, Birgegård A, Jordan J, Eating Disorders Working Group of the Psychiatric Genetics Consortium (2022). Exploring the clinical and genetic associations of adult weight trajectories using electronic health records in a racially diverse biobank: a phenome-wide and polygenic risk study. Lancet Digit Health.

[31] Chazot C (2009). Why are chronic kidney disease patients anorexic and what can be done about it?. Semin Nephrol.

[32] Musialik J, Suchecka W, Klimacka-Nawrot E, Petelenz M, Hartman M, Blońska-Fajfrowska B (2012). Taste and appetite disorders of chronic hepatitis C patients. Eur J Gastroenterol Hepatol..

[33] Ramsoekh D, Taimr P, Vanwolleghem T (2014). Reversible severe hepatitis in anorexia nervosa: a case report and overview. Eur J Gastroenterol Hepatol.

[34] El Ghoch M, Bazzani P, Dalle Grave R. Management of ischiopubic stress fracture in patients with anorexia nervosa and excessive compulsive exercising. BMJ Case Rep. 2014; bcr2014206393. 10.1136/bcr-2014-206393 PMC4195215 25301426

[35] Johnson JS, Cote AC, Dobbyn A, Sloofman LG, Xu J, Cotter L et al. The phenome-wide consequences of anorexia nervosa genes. *medRxiv* 2021; 10.1101/2021.02.12.21250941.

[36] Abdulkadir M, Hübel C, Herle M, Loos RJF, Breen G, Bulik CM (2022). The impact of anorexia nervosa and BMI polygenic risk on childhood growth: A 20-year longitudinal population-based study. Am J Hum Genet.

[37] Johansson T, Birgegård A, Zhang R, Bergen SE, Landén M, Petersen LV (2022). Polygenic association with severity and long-term outcome in eating disorder cases. Transl Psychiatry.

[38] Garber AK, Cheng J, Accurso EC, Adams SH, Buckelew SM, Kapphahn CJ (2019). Weight loss and illness severity in adolescents with atypical anorexia nervosa. Pediatrics.

[39] Forney KJ, Brown TA, Holland-Carter LA, Kennedy GA, Keel PK (2017). Defining ‘significant weight loss’ in atypical anorexia nervosa. Int J Eat Disord.

[40] Hay P, Mitchison D, Collado AEL, González-Chica DA, Stocks N, Touyz S (2017). Burden and health-related quality of life of eating disorders, including Avoidant/Restrictive Food Intake Disorder (ARFID), in the Australian population. J Eat Disord..

[41] Peebles R, Hardy K, Wilson JL, Lock JD (2010). Are diagnostic criteria for eating disorders markers of medical severity?. Pediatrics.

[42] Haelle T. Expanded clinical definition of anorexia may help more teens. Scientific American. 2013.

[43] Veillette LAS, Serrano JM, Brochu PM. What’s weight got to do with it? Mental health trainees’ perceptions of a client with anorexia nervosa symptoms. Front Psychol. 2018;9:2574. 10.3389/fpsyg.2018.02574 PMC6304369 30618990

[44] Sawyer SM, Whitelaw M, Le Grange D, Yeo M, Hughes EK. Physical and psychological morbidity in adolescents with atypical anorexia nervosa. Pediatrics. 2016;137:e20154080. 10.1542/peds.2015-4080 27025958

[45] Saloner B, Wilk AS, Levin J (2020). Community Health Centers and access to care among underserved populations: a synthesis review. Med Care Res Rev.

[46] Duncan L, Shen H, Gelaye B, Meijsen J, Ressler K, Feldman M (2019). Analysis of polygenic risk score usage and performance in diverse human populations. Nat Commun..

[47] Martin AR, Kanai M, Kamatani Y, Okada Y, Neale B, Daly MJ (2019). Current clinical use of polygenic scores will risk exacerbating health disparities. Nat Genet..

[48] Wei W, Bastarache LA, Carroll RJ, Marlo JE, Osterman TJ, Gamazon ER (2017). Evaluating phecodes, clinical classification software, and ICD-9-CM codes for phenome-wide association studies in the electronic health record. PLoS One.

[49] Casey JA, Schwartz BS, Stewart WF, Adler NE (2016). Using electronic health records for population health research: a review of methods and applications. Annu Rev Public Health.

[50] Lawman HG, Ogden CL, Hassink S, Mallya G, Veur SV, Foster GD (2015). Comparing methods for identifying biologically implausible values in height, weight, and body mass index among youth. Am J Epidemiol..

