# Supplementary material for: Genetic investigation of the contribution of body composition to anorexia nervosa in an electronic health record setting
Source: Transl Psychiatry. 2022 Nov 19;12:486. doi: 10.1038/s41398-022-02251-y (PMC9675730; doi:10.1038/s41398-022-02251-y)
Supplement: Supplementary file 1 — Supplementary Text [file 41398_2022_2251_MOESM1_ESM.docx]

**Genetic investigation of the contribution of body composition to anorexia nervosa in an electronic health record setting**

Taralynn Mack^1,2^, Sandra Sanchez-Roige^1,3^, Lea K Davis^1,2,4,5,6^

^1^Department of Medicine, Division of Genetic Medicine, Vanderbilt University Medical Center, Nashville, TN 37232, USA; ^2^Vanderbilt Genetics Institute, Vanderbilt University Medical Center, Nashville, TN; ^3^Department of Psychiatry, University of California San Diego, La Jolla, CA 92093, USA; ^4^Department of Biomedical Informatics, Vanderbilt University Medical Center; ^5^Department of Psychiatry and Behavioral Sciences, Vanderbilt University Medical Center; ^6^Department of Molecular Physiology and Biophysics, Vanderbilt University

**Supplementary Text**

*Calculation of BMI*

For each individual, raw BMI measurements were collected from each visit along with the date of the visit, demographic data and date of birth. The age of the patient at each BMI measurement was calculated using the date of birth and date on which BMI measurement was obtained. Measurements taken during pregnancy or possible pregnancy were eliminated. Individual BMI measurements were corrected for Biologically Implausible Values (**BIVs**) in height, weight, or BMI as they are likely to be mis-entries.

To clean BIVs values, we considered three methods.[^50^](https://www.zotero.org/google-docs/?qFH1J4) The first method used externally defined BMI limits from growth charts, other similar studies, or surveys to determine the appropriate constraints on BMI. However, this method is difficult to standardize, and it may fail to identify values that are not inherently biologically implausible but are biologically implausible for a particular individual. An example of such a value would be one that is in the normal range but changes too drastically from the other recorded values in a short period of time. The second method is using internally defined BMI limits, accomplished using statistical methods such as z-scores or standard deviations. However, this approach fails to eliminate BIVs for patients with few total measurements particularly when the BIV is within the z-score range for a given individual. The final approach is the use of a combination of both externally and internally defined BIVs. Usually this method is implemented by defining fixed exclusion criteria through external research, and then using internal statistical methods to evaluate BMI observations for each individual or by adjusting external limits based on the unique dataset. However, this method can still face the issue of applicability to other samples, as each population would again need a unique set of criteria.[^25^](https://www.zotero.org/google-docs/?broken=h2gZ7F) This third approach is the most effective because it includes the benefits of both the internal and external criteria. However, there was still not a standard way of incorporating both approaches that was statistically sound. Therefore, using the previous literature as a guide, we developed a new method to effectively identify BIVs.

Our approach was intended to be applicable to any sample and tailored to the particular sample characteristics. Across all cases and controls, there were a total of 3,005,791 recorded BMI observations that were included in our cleaning protocol. First, fixed exclusion criteria were set to a minimum BMI of 12 and a maximum BMI of 60, and all values outside of this range (n=1,028) were eliminated. These criteria were determined to be the constraints of biological plausibility, specifically for women. These restrictions were determined through external research on typical BMI values for women from the Center for Disease Control and the World Health Organization. To ensure that the minimum BMI cutoff of 12 did not exclude legitimate BMI values in AN cases, manual chart review was conducted and >95% of values below 12 (N= 158/19,540) were determined to be illegitimate. Next, a z-score transformation was applied to all BMI observations for each individual. Values that did not fall within +/-3 standard deviations were filtered out (n=562). This effectively removed values that were biologically plausible, but not plausible for that specific individual. Using these criteria, ~1% of measured values were eliminated. Following this process, individual mean BMI and lowest BMI were calculated for each case based on the cleaned BMI values. For cases, the average value for mean BMI was 20.95 (range= 14.02 – 35.92) and for lowest BMI was 18.07 (range=12.48 – 35.48). For controls, the average value for mean BMI was 25.95 (range= 14.11 – 51.07) and for lowest BMI was 23.09 (range= 12.19 – 50.76). Lastly, median age over the patient’s record and age at lowest BMI was calculated for each individual.
